# High-Resolution MgB_4_O_7_:Ce,Li OSL Foils for Bragg Curve Mapping in Proton Eye Therapy

**DOI:** 10.3390/ma19132751

**Published:** 2026-06-27

**Authors:** Michał Sądel, Leszek Grzanka, Jan Swakoń, Tomasz Horwacik, Damian Wróbel, Sebastian Kusyk, Piotr Płatek, Paweł Bilski

**Affiliations:** Institute of Nuclear Physics, Polish Academy of Sciences, PL-31342 Krakow, Poland; leszek.grzanka@ifj.edu.pl (L.G.); jan.swakon@ifj.edu.pl (J.S.); pawel.bilski@ifj.edu.pl (P.B.)

**Keywords:** two-dimensional (2D) radiation dosimetry, optically stimulated luminescence (OSL), proton radiotherapy, MgB_4_O_7_:Ce,Li, Gafchromic^TM^ EBT3 films

## Abstract

By using a PMMA-made therapeutic wedge and a recently developed reusable silicone foil dosimeter based on the optically stimulated luminescence (OSL) of MgB_4_O_7_:Ce,Li (MBO) material, direct measurements of the complete proton Bragg curves for two independent clinically relevant proton beams were achieved. The PMMA wedge compensator created a controlled range gradient across the beam field, enabling comprehensive characterisation of Bragg curve features, including the entrance plateau, the maximum of the Bragg peak, and the dosimetrically critical distal fall-off region. Measurements were performed using a dedicated, self-built (3D-printed) optical detection setup equipped with a blue LED (440 nm) that illuminates the MBO foil dosimeter and a highly sensitive electron-multiplication (EMCCD) camera, which simultaneously acquires 2D OSL light from the foil. The prototype technology enables single-shot 2D mapping of the complete Bragg curve. Validation against Monte Carlo (MC) simulations and Gafchromic^TM^ EBT3 films demonstrates sub-millimetre accuracy in localising the clinically critical proton parameters: peak-to-plateau, FWHM and distal fall-off. Measurements were performed for two independent therapeutic proton beams with initial energies of 58.8 and 61.1 MeV, routinely used for proton eye-beam treatments at IFJ PAN Krakow. As a proof of concept, the results demonstrate the potential of MBO-based silicone foil technology to reproduce clinically relevant Bragg-curve parameters with accuracy approaching that of the current gold standard for passive 2D dosimetry, Gafchromic^TM^ EBT3 films, while systematic differences attributable to optical diffusion, residual LET-dependent quenching, and the dual-foil junction remain to be corrected.

## 1. Introduction

Proton therapy has emerged as a premier radiation treatment modality, offering significant dosimetric advantages over conventional photon radiotherapy [1,2,3]. The characteristic Bragg curve, defined by the proton stopping power and energy-loss straggling dose, has a gradual plateau and a sharp dose maximum [4,5]. A rapid distal fall-off region follows this maximum. In eye treatments, this distal fall-off represents both the primary clinical advantage and the most challenging aspect of proton therapy from a dosimetric verification perspective, with widths typically of 1–2 mm (90–10%) in dedicated systems [6,7].

The margins for eye treatments are smaller than those used for deeper-seated tumours, typically 2–2.5 mm laterally and distally, to account for penumbra and setup uncertainties, with even tighter constraints required for larger tumours or posterior locations near critical optic structures [8,9]. For a typical 58.8–60 MeV ocular proton beam, the range in water is approximately 29 mm; the clinically applied margins of 2–2.5 mm are comparable to, or may exceed, the width of the distal fall-off region located at that range. Consequently, accurate measurement and verification of this region are essential for quality assurance (QA) [10,11], yet remain technically challenging due to the requirement for submillimeter spatial resolution.

Current dosimetric verification tools face significant limitations when measuring steep distal fall-off gradients. Ionisation chamber arrays, widely used for routine QA, typically feature detector spacing of 5–10 mm [12,13], which is insufficient to adequately sample regions where dose varies over sub-millimetre distances. Semiconductor diode arrays offer improved resolution (2.5–5 mm) but remain inadequate for resolving the finest gradient features and may exhibit an energy-dependent response [14].

Radiochromic films, particularly Gafchromic^TM^ EBT3, are considered the clinical gold standard for 2D dose verification due to their high spatial resolution (~0.1 mm) and near-tissue equivalence [15,16,17]. However, they are single-use detectors with a limited dynamic range (recommended up to ~10 Gy, saturation around 40 Gy) [18,19], require careful handling and post-processing (including proton-quenching calibration), and incur recurring costs.

The challenge becomes more pronounced when range-modifying devices such as compensators or wedges are used, as verification of complex dose distributions requires high-resolution 2D dosimetry capable of detecting millimetre-scale discrepancies between planned and delivered doses. While gel dosimeters provide quasi-tissue-equivalent responses and enable volumetric verification, their oxygen sensitivity necessitates sealed containers and readout via MRI or optical systems [20].

Optically stimulated luminescence (OSL) dosimetry has recently emerged as a promising approach for high-resolution, reusable radiation dose measurements [21,22]. In OSL materials, energy deposited by ionising radiation is stored in metastable trap states and later released as luminescence upon optical stimulation, with intensity proportional to the absorbed dose.

Recent developments have focused on embedding OSL powders in transparent silicone matrices to form flexible foils, enabling 2D dose mapping by illuminating the foil surface and imaging the emitted signal with a CCD camera [23,24]. This technique combines key advantages of radiochromic films (high spatial resolution and full 2D information) with those of OSL technology, including reusability, wide dynamic range, and rapid readout.

Jensen et al. demonstrated high-resolution 2D dose mapping [25]. They used ion-beam therapy with Al_2_O_3_:C OSL films, which offer a large detection area but lower spatial resolution than the MBO-based approach presented in this work.

The newly emerging OSL material magnesium borate doped with cerium and lithium (MgB_4_O_7_:Ce,Li, hereafter MBO) exhibits particularly favourable properties for proton therapy dosimetry. MBO demonstrates an effective atomic number (Z_eff ≈ 8.4) [26] very close to that of soft tissue (Z_eff ≈ 7.4) and significantly reduced luminescence quenching compared to other OSL materials when exposed to high-density ionising radiation [27,28]. This quenching effect—decreased luminescence efficiency with increasing linear energy transfer (LET)—has been a major limitation of OSL dosimetry in proton beams, where LET increases up to tenfold toward the distal Bragg peak [29,30]. MBO maintains a relative luminescence efficiency above 0.75—defined as the OSL response per unit dose relative to a low-LET (^60^Co gamma) reference [31]—even in the Bragg peak region, whereas previously studied materials such as LiMgPO_4_ fall below 0.60 [31,32,33,34,35,36]. Additionally, MBO exhibits minimal signal fading (<1% over 40 days), high saturation dose levels (>7 kGy), and efficient bleaching (in a limited dose range below 40 Gy) for reuse without property degradation [33,34]. While the effective atomic number of MBO is close to that of soft tissue—favourable for a photon-equivalent response—proton dosimetry is governed primarily by mass stopping power; the Bragg-peak depth and shape in the MBO/silicone matrix therefore differ from those in water, which is why all profiles are expressed on a water-equivalent-thickness (WET) axis (Section 2.6) and benchmarked against Monte Carlo simulations performed in water.

In our recent work [35], we demonstrated fabrication and characterisation of MBO silicone foils for 2D OSL proton dose mapping, achieving spatial matrix resolution of 0.074 mm/pixel—comparable to radiochromic films—and successfully measured radial dose profiles from collimated 60 MeV proton beams with agreement within 5% compared to EBT3 films. However, due to the setup configuration, measurements were performed with a step size of each foil size (a stack of MBO foils, one after another, with a 0.43 mm water-equivalent thickness, WET for a single foil) [36,37]. This means that in the distal part of the BP region, which is 1–2 mm in size, adequate measurements cannot be obtained with high spatial resolution. Consequently, the performance of MBO foils in measuring the complete Bragg curve, particularly the critical distal fall-off with its steep gradients and elevated LET, remains to be established. Moreover, validation against independent computational methods and verification of the capability to measure complex dose distributions produced by range-modifying devices, with special attention to the distal part of the Bragg peak, have not been demonstrated using 2D OSL dosimetry elsewhere. Unlike the sequential multi-foil stacking used in our previous work [35,36,37], which limits depth sampling to a single foil water-equivalent thickness, the wedge-compensator approach introduced here reconstructs the complete Bragg curve—including the steep distal fall-off—from a single irradiation at the full lateral pixel resolution of the detector, while retaining the reusability and wide dynamic range that distinguish OSL foils from single-use radiochromic films.

The present study represents a step toward future 2D validation of the entire proton Bragg curve, from the entrance plateau to the dosimetrically critical distal fall-off region, with sub-millimetre resolution (<0.1 mm). The detector provides sub-millimetre pixel sampling (0.074 mm/pixel); however, the effective spatial resolution in steep-gradient regions is currently limited by the optical point-spread function (PSF) of the readout system, which broadens the measured distal fall-off by approximately 0.5 mm relative to EBT3 and Monte Carlo references (see Section 3.2). We employed a specially prepared phantom consisting of prototype MBO foil dosimeters, which offers an extended detection area of approximately 40 mm × 20 mm, positioned after a PMMA wedge compensator. This configuration enabled comprehensive measurement of the full Bragg curve by creating a controlled range gradient across the beam field, allowing simultaneous capture of all clinically relevant regions—the entrance plateau, Bragg peak, and steep distal fall-off—within a single experimental setup. The measurements were performed for two independent proton beams with initial energies of 58.8 and 61.1 MeV (29 mm and 31.5 mm ranges in water, respectively), both dedicated to eye-beam therapy at IFJ PAN Krakow. The resulting Bragg curves from these two beams, modulated by the wedge compensator, produced a complex dose distribution that challenged the dosimetric system’s ability to accurately resolve spatial dose gradients, particularly in the steep distal fall-off regions. Measurements were cross-validated against EBT3 films and Monte Carlo simulations of the complete experimental geometry (including the wedge compensator).

## 2. Materials and Methods

### 2.1. MBO Foil Dosimeters

The MBO silicone foils used in this study were fabricated according to the procedure described in detail in our previous work [35]. Briefly, the foil manufacturing consisted of MgB_4_O_7_:Ce,Li (MBO) powder (synthesised at the Paul Scherrer Institute [31]), mixed with SYLGARD^®^ 184 silicone elastomer (Dow Corning, Midland, MI, USA) at a 1:3 weight ratio. The MBO material was doped with a nominal 0.3 mol% Ce and 10 mol% Li and prepared via solid-state synthesis at the Paul Scherrer Institute; the achieved dopant concentrations and their effect on dosimetric performance were quantitatively characterised in [33,34]. The MBO powder, sieved to a grain size below 125 µm, was homogenously mixed with the silicone matrix according to the procedure described in detail in our previous publication. The resulting circular single foil had a diameter of 20.0 ± 0.1 mm and a thickness of 0.43 ± 0.05 mm, with a water-equivalent thickness (WET) of 1.05 mm per foil.

### 2.2. Optical Detection System

The optical detection system and the readout protocol used for MBO foil were identical to those described in [35]. Images were acquired using µManager software (version 1.53c) [38], with a 30 s acquisition time per foil measurement. The raw 2D OSL images from the MBO foils were processed using custom Python (v3.13) scripts with NumPy (v2.3.4) [39], SciPy (v1.16.2) [40], and Matplotlib (v3.10.7) libraries [41]. The analysis workflow was described in detail in our previous work [35].

### 2.3. Proton Beams

To demonstrate the versatility and broad applicability of the MBO OSL dosimetry system across different proton therapy facilities and beam energies, measurements were performed using two independent proton beams from separate facilities available at the Institute of Nuclear Physics, Polish Academy of Sciences (acronym: IFJ PAN, Krakow, Poland). Absolute dose calibration for each proton beam was performed using a PTW Markus parallel-plate ionisation chamber (PTW-Freiburg, Freiburg, Germany) positioned in a water phantom. Depth–dose profiles were measured for single-beam configurations (without the wedge) to establish the reference Bragg curve characteristics and verify beam energy. A brief characterisation of the used proton beams is given below:

#### 2.3.1. Beam 1

The first proton beam was delivered from the Proton Eye Radiotherapy Facility, which utilises the AIC-144 isochronous cyclotron (IFJ PAN, Krakow, Poland) [42]. This facility provides a monoenergetic 60 MeV proton beam specifically designed for ocular tumour treatments. The beam characteristics are:

- Energy: 60.0 MeV nominal energy, 58.8 MeV at isocenter;
- Range in water: approximately 29 mm;
- Collimation: 40 mm diameter brass collimator;
- Beam delivery: passive scattering system with uniform field distribution.

#### 2.3.2. Beam 2

The second proton beam was delivered from the Eye Therapy Laboratory at the Centrum Cyklotronowe Bronowice, (CCB, Cyclotron Centre Bronowice, IFJ PAN, Krakow, Poland) [43]. Although the cyclotron at the CCB can accelerate protons to energies up to 230 MeV for full-body treatments via gantry systems, the CCB Eye Therapy Laboratory operates with a degraded beam for ocular tumour treatments. The beam characteristics are:

- Energy: 230.0 MeV nominal energy, 61.1 MeV at eye therapy room at isocenter;
- Range in water: approximately 31.5 mm;
- Collimation: 40 mm diameter brass collimator;
- Beam delivery: extracted from the experimental hall beamline with magnetic deflection to the eye therapy position;
- Energy selection: achieved through the energy selection system (ESS) degrader.

### 2.4. Gafchromic^TM^ EBT3 Film Dosimetry

Gafchromic^TM^ EBT3 radiochromic films (Ashland Inc., Bridgewater, NJ, USA) were used as the 2D dosimetry reference standard. Films were cut to 60 mm × 60 mm to determine the proton beam dimensions and were positioned behind the wedge as the MBO foils. After irradiation, the EBT3 films were stored in the dark for 24 h to allow polymerisation to stabilise. Films were scanned using an Epson Perfection V850 Pro flatbed scanner (Epson, Suwa, Japan) in RGB transmission mode at 150 DPI resolution. An in-house calibration procedure based on red-channel readout and a polynomial fit to the optical density response was employed, as detailed in our previous study [35]. The EBT3 films were irradiated with 5 Gy proton beams, within the linear response range for radiochromic film calibration. The optical density response method for the EBT3 films was also described in our earlier work [35]. The MBO foils, by contrast, were irradiated at a 20 Gy entrance dose. This higher dose level is required to achieve a sufficient signal-to-noise ratio in the OSL response, given MBO’s inherently weaker photon emission relative to the pronounced optical density change in radiochromic films. It is well within the linear dose–response range previously characterised for MBO foils [31,32]. The dose-level difference does not affect the comparison of dosimetric parameters reported in this work, as all profiles are normalised to the entrance-plateau region. Because both detectors operate within their respective linear dose–response ranges (EBT3 at 5 Gy, below the ~10 Gy recommended limit [18,19]; MBO at 20 Gy, within the linear OSL range established in [31,32,35]) and because all profiles are normalised to the entrance plateau, the difference in absolute dose does not influence the shape-based dosimetric parameters (FWHM, Max/Plateau, distal fall-off) compared in this study. 

### 2.5. Experimental Setup with Therapeutic Wedge Compensator

**Figure 1:**
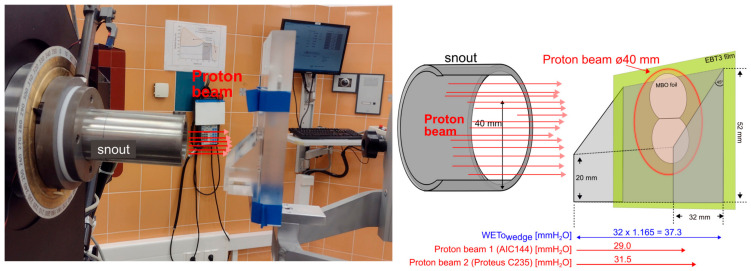
An experimental setup with a wedge compensator was used to measure the two proton Bragg peaks available from beam 1, AIC-144 (58.8 MeV, range in water 29 mm), and from beam 2, Proteus C235 cyclotron (proton energy at the eye therapy room: 61 MeV, range in water: 31.5 mm). The wedge compensator was designed and fabricated at IFJ PAN [44]. The MBO foils, protected with black tape, were mounted in the isocenter of the therapy station on the eye therapy chair in the proton treatment rooms of the two Proton Eye Radiotherapy facilities. The construction of the setup comprised the wedge compensator and the MBO foils mounted behind it. In a separate irradiation set, the EBT3 films were irradiated under the same conditions.

A PMMA (polymethyl methacrylate) wedge compensator was used to provide the proton range-modulation characteristics of the two different Bragg peaks. The wedge was previously designed and fabricated at IFJ PAN (Krakow, Poland) for the ProBImS (Proton Beam Imaging System, IFJ PAN, Kraków, Poland), developed for the QA programme of the proton beam delivered by the AIC-144 cyclotron [44,45], and is routinely used in daily QA practice at the IFJ PAN proton facility. The basic wedge material parameters are as follows: density: 1.18 g/cm^3^, WET: 1.165 (1 mm of PMMA equals 1.165 mm of water in terms of proton energy loss). Figure 1 shows the geometric specifications: a trapezoidal wedge (right-angled trapezoidal cross-section, wedge angle: 45°). The base dimensions comprise: a short base, 20 mm; a long base, 52 mm. The total thickness is 32 mm (constant across the width). The WET range modulation is as follows: a 45° wedge angle creates a range modulation of approximately 0 to 37 mm WET (expressed in water range) across the beam field (calculated as: 32 mm × 1.165 = 37.3 mm WET maximum).

During proton irradiation, the wedge compensator was mounted on the surface of the PMMA phantom holder and positioned atop the therapeutic chairs. The MBO foil and EBT3 film were placed separately (for each irradiation) behind the wedge, in an active area available to both beams (40 mm diameter proton beam). The wedge was oriented so that the gradient direction aligned with the control of proton-range propagation across the beam field. A precision-machined frame ensured reproducible wedge positioning for both beam deliveries, maintaining an identical geometric configuration for both irradiations.

The PMMA phantom, together with the wedge, was mounted on the therapeutic chair in the treatment room and aligned to the beam isocenter using the facility’s laser positioning system. The wedge orientation was carefully aligned to ensure consistent geometry for both beam deliveries. The MBO foils were covered with light-tight black tape to protect from ambient light exposure during irradiations and handling.

To accommodate the spatially extended dose distribution produced by the PMMA wedge compensator, a custom-built prototype MBO phantom was prepared for this study. Two standard circular MBO foils (each 20.0 ± 0.1 mm in diameter, 0.43 ± 0.05 mm in thickness) were trimmed along one edge and assembled with their flat-cut edges in contact, creating an extended detection area of approximately 40 mm × 20 mm (see Figure 1). This configuration enabled coverage of the complete range-modulated dose gradient induced by the wedge compensator across the beam field. The MBO foils were next covered with light-tight black tape to protect them from ambient light during mounting on the wedge compensator. Each foil retained a water-equivalent thickness (WET) of 1.05 mm. The foils were positioned in a wedge such that they extended slightly upstream of the wedge compensator (see Figure 1—MBO foils position). This positioning ensured that a portion of the foils was exposed to the unmodulated beam (before entering the wedge), allowing measurement of the Bragg peak onset region, while the remainder was exposed to the range-modulated dose distribution across varying PMMA wedge thicknesses. The separate MBO detector assemblies were used for irradiations with proton beams from different cyclotron facilities at IFJ PAN, enabling a direct comparison of the detector’s performance across clinically relevant beam energies for ocular proton therapy. The entrance dose delivered to the MBO foils was set to 20 Gy for both proton beams. A picture taken from the therapy room during measurements, together with a visualisation of the wedge and experimental setup, is shown in Figure 1.

### 2.6. Monte Carlo Particle Transport Simulations

Monte Carlo simulations were performed using the SHIELD-HIT12A (v1.1.1) particle transport code on the yaptide computation platform (v2.6.6) [https://github.com/yaptide, accessed on 25 June 2026]. The 58.8 MeV proton beam from the AIC-144 cyclotron was modelled using commissioning data from the IFJ PAN proton eye therapy facility. The simulation methodology follows the approach described in detail in our previous publication [35].

**Figure 2:**
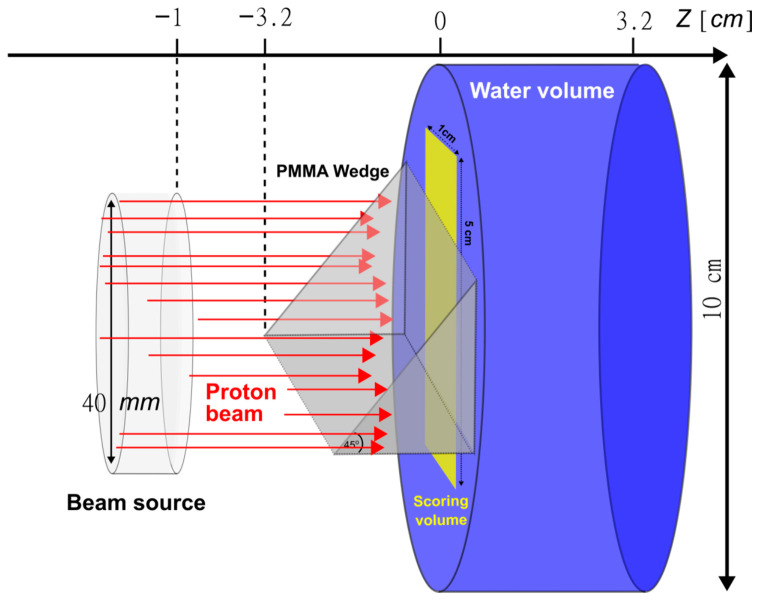
Schematic representation of the Monte Carlo simulation geometry used in this study, modelled in SHIELD-HIT12A on the yaptide platform. The proton beam (red arrows) enters through the 40 mm diameter steel collimator (snout, left) and traverses the PMMA wedge compensator before reaching the scoring region. Dose scoring was performed in a 1 mm-thick water box with a 5 × 1 cm front face (yellow rectangle).

For the Proteus C-235 beamline (CCB, Cyclotron Centre Bronowice, IFJ PAN, Krakow, Poland), the beam source was modelled with a kinetic energy of 61.1 MeV and an energy spread of σ = 0.75 MeV. The beam source was located at z = −4 cm relative to the phantom entrance, within a cylindrical water phantom with a radius of 5 cm. A schematic representation of the simulation geometry is presented in Figure 2. The proton beam (red arrows) enters through the 40 mm diameter steel collimator (snout) and traverses the PMMA wedge compensator before reaching the scoring region. The cylindrical water phantom (diameter 10 cm) contains the scoring plane (yellow rectangle) positioned at the location of the MBO foil dosimeter. The wedge compensator creates a spatially varying range modulation across the beam field (see Section 2.5), resulting in a depth-dependent dose distribution scored within the phantom volume. The geometry reproduces the experimental configuration described in Section 2.5.

Dose scoring behind the wedge was performed in a 1 mm-thick water box with a 5 × 1 cm front face (yellow rectangle in Figure 2). The scoring volume was divided into 500 bins of 0.1 mm size, matching the spatial resolution of the optical detection system. A total of 10^7^ primary protons were simulated for each beam configuration. The statistical (Monte Carlo) uncertainty of the scored dose was below 1% per 0.1 mm bin in the high-dose region; the dominant uncertainty is therefore systematic, arising from the beam-model parameters. For both the AIC-144 and C-235 beams, the simulated depth–dose curves were compared with Markus ionisation-chamber measurements obtained for the corresponding reference configurations without the wedge, showing agreement within 5% in the entrance high-dose region. This relative-dose level characterises the performance of the present prototype detector system and is not yet at the ~3% dose tolerance targeted for clinical proton therapy; reaching clinical tolerances will require the optical-PSF and LET-quenching corrections discussed in Section 3.2.

The beam model was validated by comparing simulated depth–dose distributions against Markus ionisation chamber measurements for single-beam configurations without the wedge, following the methodology established in our previous publications [35]. The proton beam range agreement between simulation and measurement was better than 5% across the full depth–dose distributions of both beam configurations, confirming the reliability of the beam source models. Having established this validated MC framework, we extended the simulations to include the PMMA wedge compensator geometry, enabling direct comparison with the MBO foil and EBT3 film measurements described in Section 2.5. This approach was applied independently for both proton beams: the 58.8 MeV beam from the AIC-144 cyclotron and the 61.1 MeV beam from the CCB Proteus C-235.

As expected from the different measurement geometries, the FWHM of the Bragg curve profiles measured behind the wedge (in the plane perpendicular to the beam axis) is systematically broader than the FWHM of the depth–dose curve measured along the beam axis without the wedge. This broadening arises from two contributions: (i) the different stopping power properties of PMMA relative to water, and (ii) the interplay between energy straggling and multiple Coulomb scattering at the 45° wedge edge, which effectively broadens the energy distribution at each transverse position behind the wedge beyond the straggling at the corresponding depth in water. Both effects are present for both beamlines and are accounted for in the MC simulations.

For comparability between MC simulations and the experimental setup, the depth axis presented in the Bragg curve plots (see Section 3.2) is given as water-equivalent thickness (WET). The conversion accounts for: (i) the air gap between the wedge exit face and the foil position (negligible WET contribution), (ii) the WET of the MBO silicone foil itself (1.05 mm, as established in [35]), and (iii) the WET of the black-tape light shield covering the foil (~0.05 mm). The PMMA wedge thickness traversed at each transverse position was converted to WET using the PMMA-to-water stopping power ratio of 1.165 at the relevant proton energies. These corrections were applied consistently to both the MC simulations and the experimental data, allowing all profiles to be directly compared on a common WET depth axis.

## 3. Results and Discussion

### 3.1. Two-Dimensional Dose Maps

**Figure 3:**
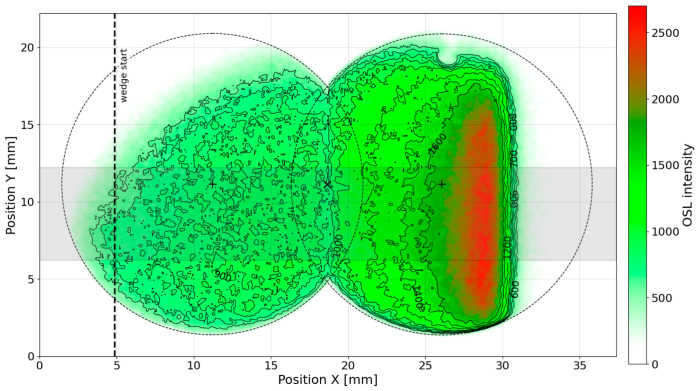
Comparison of the 2D OSL intensity obtained for the MBO foils irradiated at the AIC-144 (58.8 MeV) cyclotron with an entrance dose of 20 Gy with collimated (40 mm) proton beams. The black lines indicate the beginning of the wedge (dashed line) and the pixel area used to plot the depth profiles in Figure 6 and Figure 7 (horizontal shaded rectangle). The “+” markers indicate the centres of the two circular MBO foils forming the detector outline, and the “x” marker denotes the midpoint between them (i.e., the centre of the junction region).

**Figure 4:**
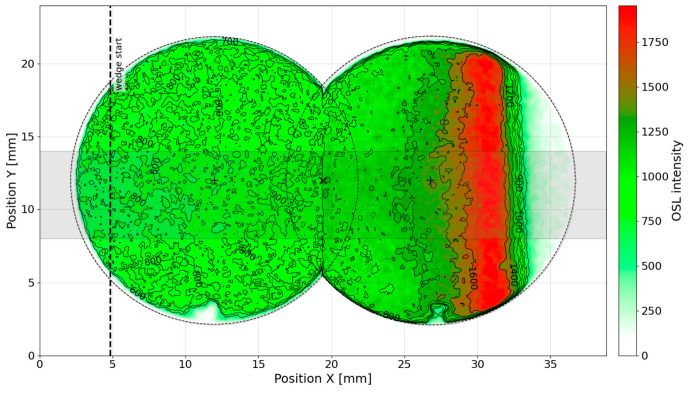
Comparison of the 2D OSL intensity obtained for the MBO foils irradiated at the Proteus C-235 (61.1 MeV) cyclotron with an entrance dose of 20 Gy with collimated (40 mm) proton beams. The black lines indicate the beginning of the wedge (dashed line) and the pixel area used to plot the depth profiles in Figure 6 and Figure 7 (horizontal shaded rectangle). The “+” markers indicate the centres of the two circular MBO foils forming the detector outline, and the “x” marker denotes the midpoint between them (i.e., the centre of the junction region).

Figure 3 and Figure 4 present the 2D maps acquired from the MBO foils and the corresponding EBT3 film images.

The 2D distributions exhibit the characteristic gradient produced by the PMMA wedge compensator: a uniform entrance region where the beam passes through minimal wedge material, followed by a progressively amplified region corresponding to increasing wedge thickness, and finally a steep drop at the distal end of the Bragg peak. The spatial extent of the modulated gradient is consistent with the theoretical WET calculated for the 45° wedge geometry across the beam field (see Section 2.5).

**Figure 5:**
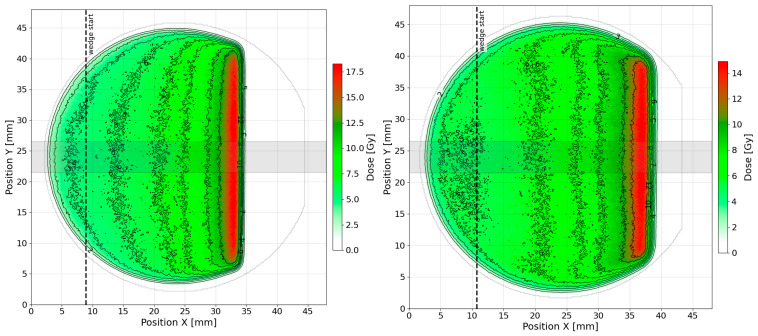
Comparison of the 2D dose–response curve obtained for the EBT3 films irradiated at the (a) AIC-144 (58.8 MeV) and (b) Proteus C-235 (61.1 MeV) cyclotrons with an entrance dose of 5 Gy with collimated (40 mm) proton beams. The black lines indicate the beginning of the wedge (dashed line) during proton irradiations and the pixel area used to plot the depth–dose profiles in Figure 6 and Figure 7 (horizontal shaded rectangles).

The entrance-plateau region of the 2D maps (Figure 3, Figure 4 and Figure 5) is laterally uniform to within ±5% near the central axis, consistent with the passive-scattering field specification; readout reproducibility of the MBO foils was established within ~1% in our previous work [35].

The region between the two cropped MBO foil segments (the junction zone, approximately 1 mm wide) appears as a decrease in signal intensity in the intensity map and was excluded from the profile analysis, as OSL powder inhomogeneities at the trimmed edge distort the local signal (see also the discussion in Section 3.2).

The dual-foil junction (~1 mm, excluded from analysis) is a limitation of the current prototype assembly. Its impact is position-dependent: negligible for the AIC-144 beam, where it lies in the entrance plateau, but non-negligible for the C-235 beam, where it coincides with the Bragg peak and contributes to the underestimated Max/Plateau ratio. A single, larger-area MBO foil—feasible with the existing fabrication procedure—would remove this junction artefact entirely in future work.

**Figure 6:**
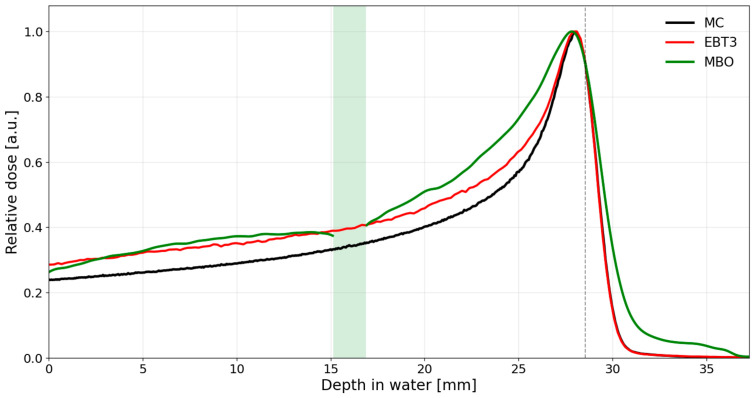
Comparison of Bragg curve profiles measured perpendicular to the beam direction for the AIC-144 beam (58.8 MeV) using MBO foils (green), EBT3 films (red), and MC simulations (black). Profiles are normalised to the maximum value. The shaded band indicates the junction zone between the two MBO foil segments. Dosimetric parameters extracted from these profiles are summarised in Table 1. Note that the Monte Carlo profile closely overlaps the EBT3 profile in the distal fall-off region, reflecting the excellent agreement between the independent simulation and the radiochromic-film reference; the MC line is plotted on top with increased weight for visibility.

Qualitatively, the MBO foil intensity images (Figure 3 and Figure 4) reproduce the key spatial features visible in the EBT3 reference films (Figure 6): the position of the Bragg peak region and the onset of the distal fall-off are consistent between the two detector types for both beams. A detailed quantitative comparison is presented in Section 3.2. 

The 2D maps presented in Figure 4 and Figure 5 include iso-signal contour lines overlaid at arbitrary signal values (in steps of 100–200) to visualise signal features and detector inhomogeneities better. Their smoothness and continuity across the MBO foil images—including at the 90% and 80% levels in the dosimetrically critical distal fall-off region—confirm that the sub-millimetre spatial resolution established in is maintained in the extended dual-foil configuration used here.

For comparison, iso-signal contours are overlaid also on the EBT3 film images (Figure 5), derived from the red-channel optical density calibration procedure described in Section 2.4. The isodose lines in the EBT3 images appear visually sharper in the distal fall-off region, consistent with the absence of lateral optical diffusion in the film and with the EBT3 scanner resolution of ~0.169 mm/pixel at 150 DPI. Despite this, the positions of the clinically relevant 90% and 80% contours in the distal fall-off are in close spatial agreement between MBO foils and EBT3 films for both beam energies, as confirmed quantitatively in Section 3.2.

Additionally, a slight reduction in signal is observed at the borders where foil cutting was applied. This is attributable to an imbalance in lateral OSL light scattering at the trimmed edge: OSL photons generated near the cut boundary preferentially travel toward the open foil interior, reducing the signal collected at that edge. A ~1 mm exclusion margin on each cut side effectively eliminates this artefact from the profile analysis. The same lateral light diffusion mechanism is responsible for the systematic broadening of the distal fall-off profile measured by the MBO foils, as discussed quantitatively in Section 3.2.

Because proton energy deposition is strongly non-uniform, repeated irradiation could in principle induce spatially heterogeneous sensitivity changes across the foil. Such variations can be detected and corrected by periodic uniform-field (flood) irradiations that map the per-pixel sensitivity, with each measurement normalised to this flood-field response—analogous to flat-field correction in imaging detectors. Long-term detector performance, including aging over many irradiation/bleaching cycles, was not investigated here; while reusability and low fading have been demonstrated for MBO foils in [33,34,35], a dedicated longevity study under repeated clinical-level irradiation is required before routine reuse can be recommended.

Figure 5 presents the corresponding 2D dose distributions recorded with EBT3 radiochromic films for both proton beams. The EBT3 images served as the primary reference standard for validating the MBO foil measurements, owing to the well-established dosimetric characteristics of EBT3 films in proton beams. The film images clearly resolve the full dose gradient introduced by the wedge compensator, with the entrance plateau, Bragg peak region, and steep distal fall-off all visible within the detector area. The horizontal black lines mark the pixel rows used for extracting the Bragg curve profiles presented in Section 3.2; the vertical dashed black line indicates the proximal start of the wedge. The most visible difference between the MBO foil images and EBT3 films is a sharper distal fall-off gradient in the EBT3 images, attributable to lateral optical diffusion of OSL light within the foil matrix. This effect is quantified and discussed in Section 3.2.

### 3.2. Bragg Curve Profiles: Comparison of MBO Foils, EBT3 Films, and Monte Carlo Simulations

**Figure 7:**
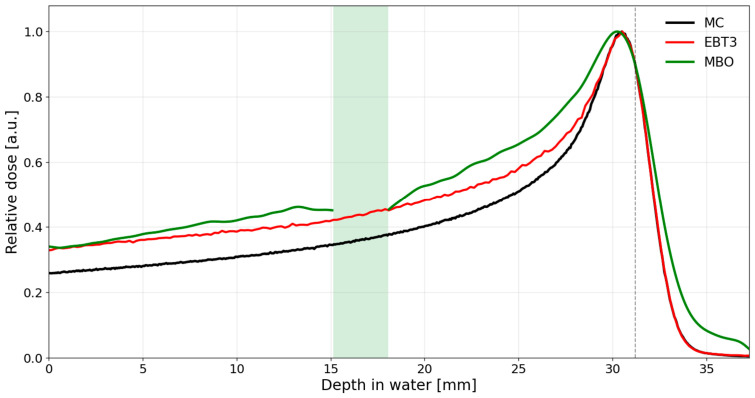
Comparison of Bragg curve profiles measured perpendicular to the beam direction for the CCB Proteus C-235 beam (61.1 MeV) using MBO foils (blue), EBT3 films (black), and MC simulations (red). Profiles are normalised to the maximum value. The shaded band indicates the junction zone between the two MBO foil segments. Dosimetric parameters extracted from these profiles are summarised in Table 2. Note that the Monte Carlo profile closely overlaps the EBT3 profile in the distal fall-off region, reflecting the excellent agreement between the independent simulation and the radiochromic-film reference; the MC line is plotted on top with increased weight for visibility.

Figure 6 and Figure 7 present Bragg curve profiles extracted perpendicular to the beam direction for the AIC-144 (58.8 MeV) and CCB Proteus C-235 (61.1 MeV) beams, respectively, comparing MBO foil measurements, EBT3 films, and MC simulations. All profiles were normalised to the maximum value. The quantitative dosimetric parameters extracted from these profiles are summarised in Table 1 and Table 2 for the AIC-144 and C-235 beams, respectively.

*Distal fall-off (80–20%).* The distal fall-off width showed consistent agreement between EBT3 films and MC simulations: for AIC-144, 0.92 mm (EBT3) vs. 0.93 mm (MC); for C-235, 1.28 mm (EBT3) vs. 1.28 mm (MC). The MBO foils yielded broader values of 1.42 mm (AIC-144) and 1.76 mm (C-235), corresponding to an overestimation of approximately 0.50–0.51 mm relative to EBT3 and MC. This systematic broadening is attributed to lateral optical diffusion within the MBO silicone foil matrix: OSL photons generated at the steep dose gradient travel laterally within the foil before being collected by the EMCCD camera, effectively blurring sharp spatial features. A similar effect was noted in our previous work [35]. Importantly, the magnitude of this broadening (~0.5 mm) is comparable to or smaller than the clinical range uncertainty margins of 1 mm typical for proton eye treatments [6,7], suggesting that MBO foil measurements remain clinically informative even without correction for optical blurring.

A quantitative correction of this broadening would require experimental determination of the effective point-spread function (PSF) of the full readout chain, including both lateral light spreading within the MBO/silicone foil and blur introduced by the optical imaging system; in the present prototype the measured profile is the convolution of the true deposited-dose distribution with this PSF. The PSF can be characterised from dedicated calibration irradiations (e.g., knife-edge exposures giving the edge- and line-spread functions), after which the measured 2D signal map could be corrected by regularised deconvolution (e.g., the Wiener or Richardson–Lucy method). The development and validation of such a procedure were beyond the scope of the present proof-of-concept study and are planned as a separate, dedicated investigation.

*Peak width (FWHM).* The full width at half maximum of the Bragg peak was consistently broader in MBO foil measurements compared to both EBT3 and MC: for AIC-144, MBO FWHM = 8.44 mm vs. EBT3 = 6.57 mm and MC = 4.89 mm; for C-235, MBO = 11.43 mm vs. EBT3 = 9.26 mm and MC = 6.44 mm. The systematic overestimation by the MBO foils (~28% for AIC-144, ~43% for C-235, all relative to EBT3) arises from the same lateral diffusion mechanism in the optical readout system responsible for the distal fall-off broadening. The larger discrepancy for the C-235 beam is consistent with the broader physical Bragg peak at 61.1 MeV, which exhibits a larger interface gradient for lateral light diffusion.

*Bragg peak-to-plateau ratio (Max/Plateau).* The peak-to-plateau ratio was consistent between MBO foils and MC for the AIC-144 beam (2.97 vs. 3.76), with EBT3 yielding a value of 3.07. For the C-235 beam, the MBO foil showed a reduced Max/Plateau of 2.59 compared to MC (3.51) and EBT3 (2.74). This discrepancy is likely influenced by interpolation at the foil junction, which lies close to the Bragg peak position for this beam configuration, and by residual LET-dependent luminescence quenching in MBO at the Bragg peak for the 61.1 MeV beam [27,28].

Although MBO shows substantially reduced LET-dependent luminescence quenching compared to previously used LiMgPO_4_ and other established OSL materials [36,37], it remains at the Bragg peak, where the LET reaches values above ~10 keV/μm. The LET-weighted luminescence efficiency of MBO, characterised in [27], decreases by approximately 5–10% at proton LET values typical of the distal Bragg peak region. For the AIC-144 beam (58.8 MeV), the foil junction lies in the entrance plateau, and the lower stopping power produces a less pronounced quenching effect, resulting in better Max/Plateau agreement with MC. For the C-235 beam, the foil junction coincides with the Bragg peak position, leading to a reconstructed signal that under-represents the true peak amplitude—the interplay of these two effects (junction geometry and residual LET quenching) therefore explains why the C-235 Max/Plateau discrepancy is more pronounced than for AIC-144.

A dedicated, spatially resolved LET measurement is not possible in the present wedge geometry, which modulates range rather than LET. However, using the η(LET) dependence characterised for MBO in [27,35] together with Monte Carlo-derived LET spectra—following the methodology described in detail in our previous study [37]—the expected luminescence-efficiency loss in the Bragg-peak and distal fall-off region is approximately 5–10%, consistent with the observed Max/Plateau discrepancy for the C-235 beam. A dedicated, in-depth LET analysis would allow this efficiency drop to be corrected; its detailed implementation, applied to the present wedge configuration, was beyond the scope of this work and is planned as the subject of a separate, future publication.

### 3.3. Two-Beam Comparison

**Figure 8:**
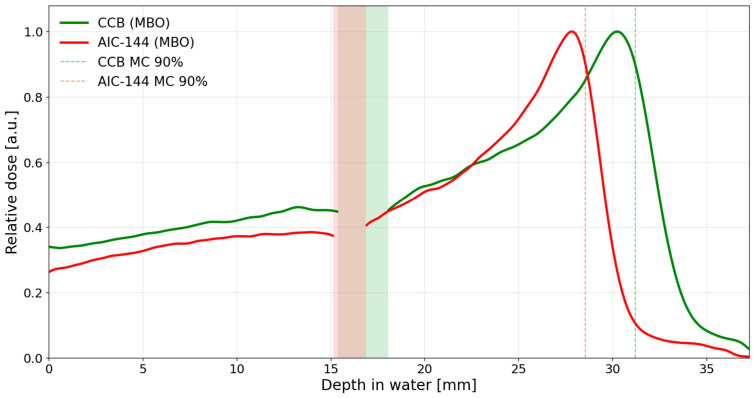
Overlay comparison of relative Bragg curve profiles measured with MBO foils for the two proton beams: AIC-144 at 58.8 MeV (red) and Proteus C-235 at 61.1 MeV (green). Both profiles are normalised to the maximum value and aligned to match the respective range as calculated using the MC method (see Section 2.6 and Figure 6 and Figure 7). The colour bands indicate the junction zone between the two foil segments.

Figure 8 presents a direct overlay of the Bragg curve profiles measured with MBO foils for both 58.8 MeV and 61.1 MeV proton beams, normalised to the maximum value and aligned at the wedge front. The broader FWHM and distal fall-off of the C-235 profile relative to AIC-144 are consistent with the higher initial energy and correspondingly larger energy straggling at depth. This comparison demonstrates the prototype MBO foil dosimeter’s capability to discriminate clinically relevant energy differences (~2 MeV) between two independent proton eye therapy beams in a single-shot measurement, without stacking multiple foils (as we presented previously) or ionisation chambers. The broader FWHM and distal fall-off of the C-235 profile relative to AIC-144 are consistent with the higher initial energy and correspondingly larger energy straggling at depth (passive-scattering system vs. the energy-selection system (ESS) degrader for C-235).

## 4. Conclusions

MgB_4_O_7_:Ce,Li (MBO) OSL silicone foils were demonstrated in a single-shot wedge-compensator configuration for complete proton Bragg curve mapping—from the entrance plateau through the peak to the steep distal fall-off region. The wedge compensator geometry enabled simultaneous acquisition of the full Bragg curve in a single irradiation, overcoming the spatial-resolution limitation inherent in sequential multi-foil stacking approaches. The present evaluation is intentionally limited to the low-energy beams used in ocular proton therapy (58.8 and 61.1 MeV), for which the detector field size and the clinically critical distal fall-off are most relevant; extension to higher beam energies, spread-out Bragg peaks, and pencil-beam scanning will be required before the method can be generalised to broader proton therapy applications.

The measurements were conducted for two independent clinical proton beams at IFJ PAN Krakow (58.8 MeV from AIC-144 and 61.1 MeV from CCB Proteus C-235). The distal fall-off width was systematically overestimated by ~0.5 mm, partially due to lateral optical diffusion within the MBO foil, a characterizable effect attributable to light scattering in the silicone matrix. In the optical readout system, the 2 cm diameter foil is located 13 cm from the CCD sensor, which introduces notable light scattering. That effect could be mitigated, but that would require a careful redesign of the readout system. On the contrary, the EBT3 films are being read out at close range using a commercial scanner, reducing the influence of light scattering. Due to the emission and absorption of spectral characteristics, such a scanner layout is not suitable for MBO foils.

The peak-to-plateau ratio showed good consistency with MC for the AIC-144 beam, with larger deviations at the C-235 energy, attributable to foil-junction geometry and residual signal quenching. These effects represent a known limitation for applications requiring precise measurement of the absolute fall-off gradient width.

Two recently published studies using alternative reusable OSL films provide useful context. Jensen et al. [25] achieved joint dose and LET mapping with Al_2_O_3_:C films over extended fields, complementing the higher spatial resolution of the MBO approach. Caprioli et al. [46] characterised a BaFBr:Eu^2+^ storage-phosphor detector—a material class well established for photostimulated-luminescence image plates [47]—across proton, carbon-ion, and electron beams, reporting Bragg peak signal quenching of up to 1.2× for clinical pencil-beam proton energies. By contrast, MBO foils demonstrated a luminescence efficiency remaining above 0.75 throughout the entire Bragg peak region at 58.8 MeV [35]—a result obtained at lower proton energy, and hence higher Bragg peak LET, than the lowest energy investigated by Caprioli et al., indicating favourable quenching properties for eye therapy beam energies. The BaFBr:Eu^2+^ film offers a larger active area (>20 × 20 cm^2^) suited to IMPT verification, while MBO foils cover the 20 × 40 mm field of proton eye therapy and support hypofractionated dose levels up to ~13 Gy—beyond the 8 Gy range investigated by Caprioli et al.

As a proof of concept, these results show that MBO silicone foils reproduce the principal Bragg-curve features (peak-to-plateau, FWHM, distal fall-off) with accuracy approaching that of the EBT3 gold standard, although systematic differences remain that are attributable to three identified and characterisable mechanisms—optical point-spread broadening, residual LET-dependent quenching, and dual-foil junction reconstruction—each amenable to the corrections outlined above. Their performance in patient-specific quality-assurance scenarios with clinical dose distributions remains to be established in future work. Taking into account previously published data showing its dynamic dosimetry range, reusability, and sub-millimetre spatial resolution comparable to radiochromic films [35], the wedge-based single-shot approach significantly simplifies the experimental protocol for complete Bragg curve measurement, demonstrating technological potential for direct application to quality assurance of proton eye therapy beams. Future work will focus on characterising the optical point spread function of the readout system to enable deconvolution of the lateral diffusion contribution and further improve agreement with reference dosimeters in the distal fall-off region.

## Figures and Tables

**Table 1:** Parameter summary for the AIC-144 cyclotron (FWHM, Max/Plateau, Penumbra 80–20%) presented separately for the EBT3 films, MC, and MBO foils.

	FWHM [mm]	Max/Plateau	Distal Fall-Off (80–20%) [mm]
**EBT3**	6.57	3.07	0.92
**MC**	4.89	3.76	0.93
**MBO**	8.44	2.97	1.42

**Table 2:** Parameter summary for the Proteus C235 cyclotron (FWHM, Max/Plateau, Penumbra 80–20%) presented separately for the EBT3 films, MC, and MBO foils.

	FWHM [mm]	Max/Plateau	Distal Fall-Off (80–20%) [mm]
**EBT3**	9.26	2.74	1.28
**MC**	6.44	3.51	1.28
**MBO**	11.43	2.59	1.76

## Data Availability

The original contributions presented in this study are included in the article. Further inquiries can be directed to the corresponding author.
